# Probing Medin Monomer Structure and its Amyloid Nucleation Using ^13^C-Direct Detection NMR in Combination with Structural Bioinformatics

**DOI:** 10.1038/srep45224

**Published:** 2017-03-22

**Authors:** Hannah A. Davies, Daniel J. Rigden, Marie M. Phelan, Jillian Madine

**Affiliations:** 1 Institute of Integrative Biology, University of Liverpool, Biosciences Building, Crown Street, L69 7ZB, UK

## Abstract

Aortic medial amyloid is the most prevalent amyloid found to date, but remarkably little is known about it. It is characterised by aberrant deposition of a 5.4 kDa protein called medin within the medial layer of large arteries. Here we employ a combined approach of *ab initio* protein modelling and ^13^C-direct detection NMR to generate a model for soluble monomeric medin comprising a stable core of three β-strands and shorter more labile strands at the termini. Molecular dynamics simulations suggested that detachment of the short, C-terminal β-strand from the soluble fold exposes key amyloidogenic regions as a potential site of nucleation enabling dimerisation and subsequent fibril formation. This mechanism resembles models proposed for several other amyloidogenic proteins suggesting that despite variations in sequence and protomer structure these proteins may share a common pathway for amyloid nucleation and subsequent protofibril and fibril formation.

The most common form of localised amyloid occurs in the aortic media (aortic medial amyloid; AMA) and is estimated to occur in 97% of Caucasian people above the age of 50^1^. The pathological impact of AMA is unknown, but it is believed that amyloid accumulation contributes to age-related diminished elasticity of the vessels and that prefibrillar intermediates of medin may underlie the pathogenesis of sporadic thoracic aortic aneurysm^2^. The main constituent of AMA is a 50 amino acid polypeptide medin, thought to be cleaved from lactadherin^3^. Molecular information regarding the structure of medin, the mechanisms that trigger medin aggregation and the formation of the insoluble fibrils is not currently available.

Amyloid formation occurs through an assembly pathway often described simplistically in successive stages of nucleation followed by elongation^4^. The nucleation point represents the trigger for progression from a ‘non-amyloid or native’ conformation into an ‘amyloid-prone’ conformation. This transition is therefore a key event in the conversion from a functional protein into a pathologically relevant state that is responsible for disease initiation and progression. Molecular information about amyloid proteins in their monomeric, intermediate or fibrillar form and regarding their interaction and aggregation to form the insoluble fibrils is sparse. This is because amyloid proteins are notoriously difficult to study in their soluble forms due to their inherent propensity to aggregate along with the heterogeneous and transient nature of the key soluble intermediates. Structural bioinformatics techniques of modelling and molecular dynamics (MD) simulations provide tools to investigate the structure and stability of these critical protein species and were employed here to generate a model for the monomeric structure of medin.

Several approaches including NMR, have been applied to probe soluble structures and the role of intermediate conformations for a range of amyloid proteins. NMR is an invaluable tool to probe individual atomic nuclei in a macromolecule in solution and can be applied to investigate the molecular structure, dynamics and kinetics of proteins. To date, many solution state studies of amyloid proteins have utilised truncated constructs^5^,^6^ and/or non-physiological conditions, often employing structure-inducing additives, detergents or lipids which often contradict or bear little resemblance to structures thought to exist in the cell^7^,^8^,^9^,^10^,^11^. However recent advances in NMR techniques are enabling more studies to be carried out on these poorly tractable, but extremely biologically important aggregating systems^12^. In this work we used direct-detected ^13^C NMR to probe soluble medin structure employing conditions that are more physiologically relevant, avoiding stabilising components. The benefits of utilising ^13^C-direct detect NMR in this study are threefold; enabling backbone assignment in shorter experimental time than conventional ^1^H-detect triple resonance experiments, larger chemical shift dispersion and shorter transverse relaxation rates that enable the observation of resonances that relax too fast in conventional ^1^H-detect experiments. Although not providing data sufficient for 3D structure determination, the experiments supported a strong fold prediction resulting from *ab initio* structure modelling.

Previous literature on medin has identified the C-terminal portion of medin, in particular residues 42–49 as highly amyloidogenic. Short peptides composed only of these eight residues are capable of rapid aggregation into microcrystalline fibres^3^,^13^. Furthermore, removal of this C-terminal region from medin abolishes amyloid forming potential^14^. It is essential to identify and understand the interactions that occur early in the aggregation process that result in formation of amyloid fibrils. Here we use a combined experimental and computational approach to elucidate the early stages of medin nucleation, information that is critical for understanding of the initiation and progression of AMA and protein aggregation in general.

## Results

### *Ab initio* modelling

**Figure 1:**
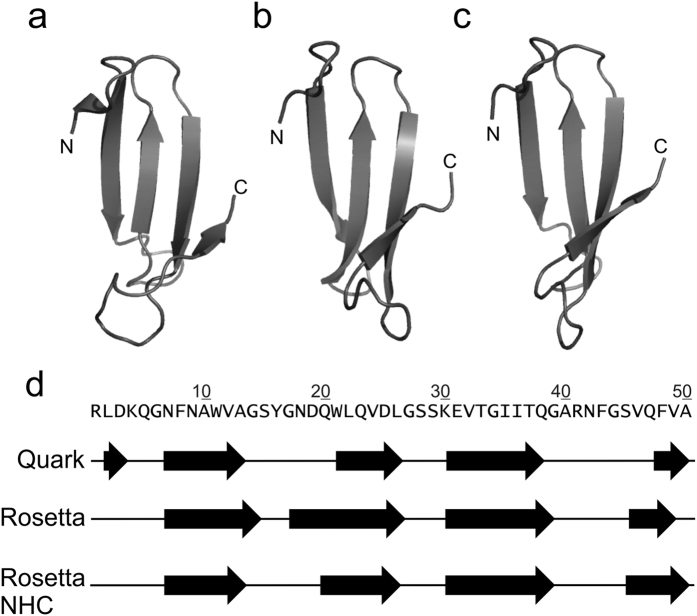
*Ab initio* fragment assembly protein modelling of human monomeric medin. Medin models generated online using QUARK (**a**) and locally in two runs of ROSETTA including (**b**) or excluding (**c**) fragments deriving from structures of lactadherin C2 domain and homologues (NHC). β-sheet positioning in the three models (**d**). Secondary structure assignment was performed using DSSP^64^ as throughout this work.

Models were generated online using QUARK^15^ and locally in two runs of ROSETTA^16^ using the sequence for medin (Fig. 1) obtained from UniProt^17^ (ID:Q08431). The QUARK and ROSETTA methods are broadly comparable in overall methodology, each assembling tertiary structures from structure fragments derived from the PDB, but differ significantly in some aspects such as the fragment libraries, which are fixed in length 3- and 9-residues in ROSETTA but are variable in QUARK and in their scoring functions. Consistency between the two methods would therefore enhance confidence in the results. The two ROSETTA runs varied in their fragment libraries: in one, fragments of full length lactadherin C2 domain crystal structures and homologous sequences were excluded (Fig. 1c, NHC). Although fragment assembly methods are based on a correlation between sequence and local secondary structure, we wished to test whether similar results were obtained when fragments of C2 domains - in which medin sequence is found in full length lactadherin - were excluded from the model building, thereby avoiding any bias towards the local conformations adopted by medin in its C2 domain context.

The results of all three modelling efforts agree to a remarkable degree (Fig. 1) and are also consistent, as expected, with secondary structure predictions (Fig. S1). Figure 1 shows the most favoured model returned by QUARK (a) alongside representatives of the largest clusters from the ROSETTA runs (b and c). In each, medin forms a four-stranded β-sheet (d). Terminal regions vary somewhat in conformation but the central portion from residues 21–35 consistently folds into a β-hairpin. This hairpin is also present in all the returned QUARK models, as well as smaller, less-favoured clusters of structures from ROSETTA modelling despite differences in terminal regions and is therefore a strongly predicted feature of the models. We have previously suggested that a similar β -hairpin may be present in insoluble medin fibrils^18^. Searches of the PDB revealed the nearest structural neighbours of QUARK and ROSETTA models to be small 3- or 4-stranded β-sheet structures in, respectively, the WW domain of human PRPF40A and *Bacillus subtilis* YmzC protein (Fig. S2). The folding pathways of WW domains have been extensively studied and implicated in amyloid protofibril generation^19^,^20^,^21^,^22^. A similar triple strand monomer structure has also been observed in MD simulations on amyloid-β^23^,^24^ suggesting that this strand arrangement may be conserved across different amyloid-prone proteins.

### ^13^C-direct detection NMR

**Figure 2:**
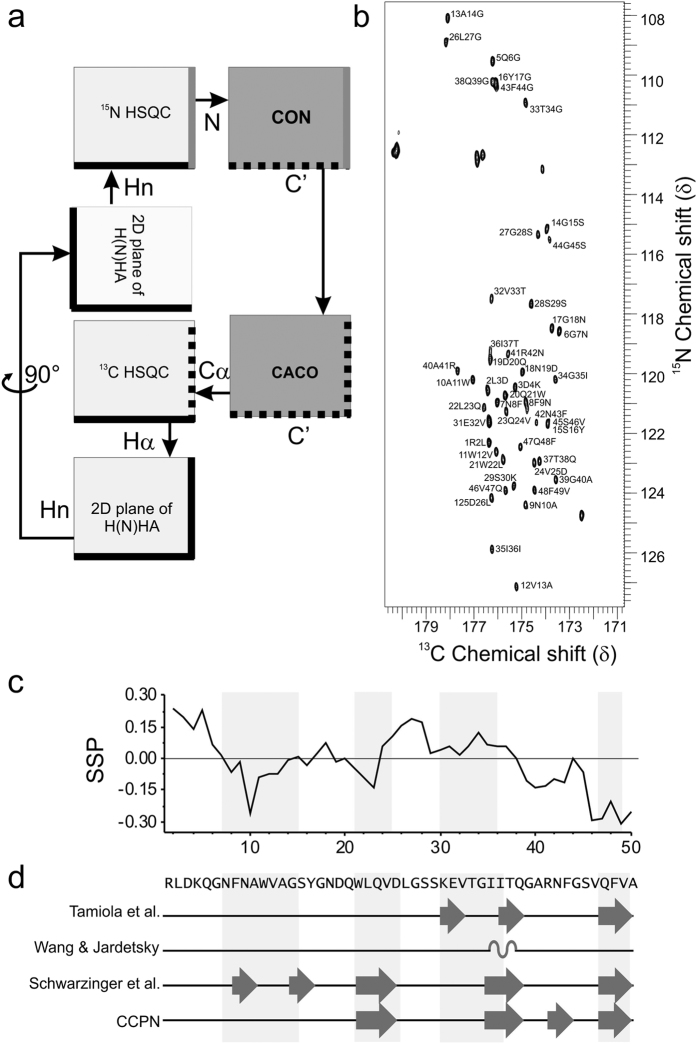
NMR data overview. (**a**) Schematic representation of the NMR workflow incorporating the use of ^13^C-direct detection experiments. (**b**) CON spectrum of human medin collected at 288 K on a Bruker Avance III NMR spectrometer operating at 14.1T using a TXO probe. (**c**) SSP^27^ output using C’ and Cα shifts for medin. (**d**) Structural propensity of backbone ΔC’, ΔCα and ΔHα chemical shifts towards either α-helix or β-strand from random coil calculated using ncIDP with reference chemical shift libraries as shown (Tamiola *et al*.^28^, Wang and Jardetsky^76^, and Schwarzinger *et al*.^77^) and CCPN^29^. Structural propensities of 10% or more are shown and summarised in Table S2. Light grey regions indicate areas of secondary structure from computational modelling as shown in Fig. 1.

Fragment assembly *ab initio* modelling methods have developed to a point that their overall performance on small proteins is good^25^ yet the reliability of their results varies and final model quality is not as predictable as homology modelling. Validation of key model characteristics was therefore sought using solution state NMR. Due to the highly dynamic and often rapid aggregation kinetics of amyloid proteins, obtaining structural information about the soluble early stages of aggregation presents a considerable challenge. In this study we employed a variety of measures to obtain the first structural information about medin in its soluble form. Previously we assigned H-N backbone resonances of medin using a combination of fast-pulsing NMR experiments and 3D triple resonance experiments acquired under denaturing conditions^26^ but were unable to assign the other backbone atoms required to elucidate secondary structure in non-denaturing conditions. Conventional 3D experiments were unfeasible due to the high protein concentration required to maintain sensitivity during a 3D pulse sequence, (under these concentrations medin aggregates rapidly within a few hours) coupled with the coherence transfer efficiency required in the longer coherence transfers of 3D pulse sequence. In contrast, ^13^C-direct detection experiments use shorter pulse sequences to reduce the loss of signal from low concentration samples. In addition the length of time required for acquisition at sufficient resolution for chemical shift dispersion between ^13^C signals, even using fast-pulsing methods was unobtainable due to the inherent instability of medin in its soluble form. As an alternative we sought to reduce dimensionality (and thus coherence transfer steps) and increase chemical shift dispersion using 2D ^13^C direct detection experiments (Fig. 2a), enabling the use of lower protein concentrations and increasing the available window of study before aggregation. Moreover, all experiments were collected at 288 K to further slow the aggregation process and extend the acquisition window. SOFAST ^1^H^15^N HMQC experiments were interleaved throughout all experiments to monitor aggregation (Fig. S3).

CON and CACO experiments yielded good chemical shift dispersion with adequate signal/noise ratios (Fig. 2b). Using this strategy it was possible to assign 98% N_H_, 98% H_N_, 98% C’, 96% Cα and 94% Hα shifts (see BMRB ID: 27021). These shifts were used to probe secondary structure propensities using Secondary Structure Propensity (SSP)^27^, neighbour corrected Intrinsically Disordered Protein Library (ncIDP)^28^ and the Collaborative Computing Project for NMR (CCPN) software Analysis^29^. SSP provides a score for secondary structure propensity for carbon shifts with negative values indicating β-strand and positive values α-helix. The backbone chemical shifts of medin indicate three areas of β-strand (residues 8–14, 21–23 and 39–50) with the C-terminal region suggesting up to 30% structural propensity (Fig. 2c). Deviation in chemical shift for medin when compared to random coil values from different chemical shift libraries including those which correct for sequence specificity was calculated using ncIDP. Structural propensities greater than 10% were observed from the ΔC’, ΔCα and ΔHα chemical shifts, with ΔN_H_ and ΔH_N_ not considered due to the susceptibility of HN chemical shifts to be sensitive to temperature^30^ and thus may produce spurious deviations from library values (Fig. 2d and Table S2)^31^. In addition, CCPN analysis of secondary structure propensity was also carried out (Fig. 2d). Overall the secondary structure analysis suggests medin has a propensity for β-strand elements (Fig. 2c,d) in good agreement with the positioning of the strands in the *ab initio* models (Fig. 1).

**Figure 3:**
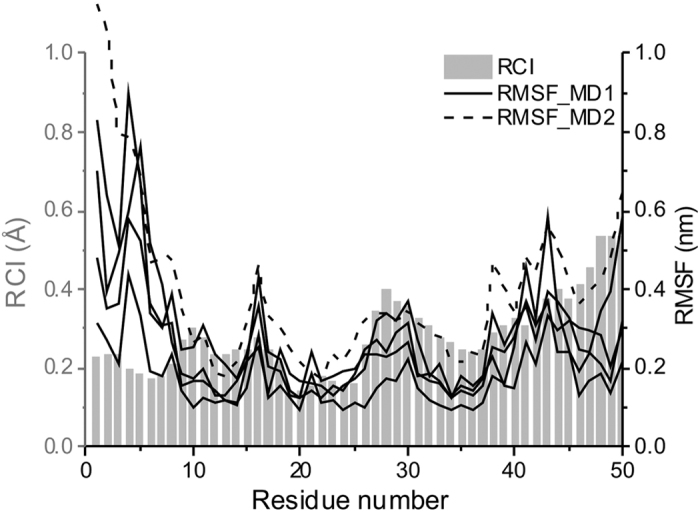
Comparison of medin backbone dynamics using random coil index and root mean square fluctuation. All atom RMSF data from OPLS-AA/L force field MD simulations (four independent trajectories)(MD1) and AMBER99SB force field with the TIP3P water model (MD2) performed on the top QUARK *ab initio* model are shown as black lines, solid and dashed respectively. RCI data derived from the NMR chemical shift data using the online server CSI 2.0^33^.

Chemical shift data was used to investigate the flexibility of the protein backbone using the random coil index (RCI)^32^ within the CSI 2.0 server^33^. Results showed increased flexibility in regions centred on residues 10 and 30, and at the C-terminus (Fig. 3). MD simulations performed on the QUARK model also indicated increased flexibility at the C-terminus and around residue 30; however, the root mean square fluctuations (RMSF) profiles are dominated by flexibility at the N-terminus which is not reflected in the RCI calculated from the NMR data (Fig. 3). It should be noted that the RCI values for the N-terminal region must be considered intrinsically less reliable than those of the rest of the protein due to the relative paucity of assignments in this region (BMRB ID: 27021).

### Molecular Dynamics simulations

**Figure 4:**
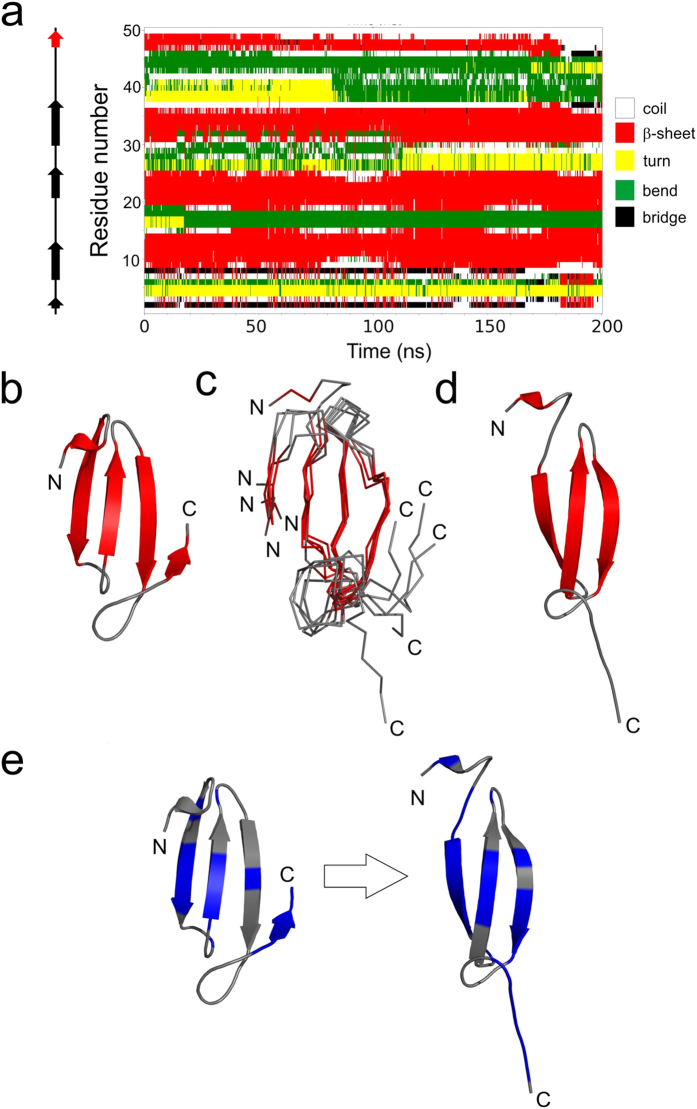
Instability of the C-terminal strand is predicted to trigger amyloidogenesis. (**a**) Secondary structure matrix of the initial OPLS-AA/L force field MD trajectory in which C-terminal strand detachment was observed. Snapshots sampled every 250ps are colour coded according to secondary structure as assigned by DSSP. Arrows on the left indicate secondary structure (β-strands) present in the starting model. (**b**) Starting QUARK model used in the simulations. (**c**) Ensemble of structures at 185, 310, 400, 600 and 900 ns from the MD simulation shown in (**a**) in which the C-terminal strand is detached from the central sheet. (**d**) Snapshot of medin at 185 ns illustrating loss of the C-terminal strand. (**e**) Per-residue aggregation propensity prediction data for the starting model (left) and the 185 ns snapshot (right) generated by Aggrescan3D^34^. Blue indicates higher aggregation propensity (>0), and grey lower values (<0).

We used MD simulations with the OPLS-AA/L force field and the SPC/E water model to investigate the stability of the medin model, the potential role of the C-terminus in amyloid formation and to propose a possible mechanism for amyloid initiation. Four independent MD simulations were ran using GROMACS (Fig. 3, solid lines). They indicated good overall model stability with the three central β-strands persisting throughout all simulations as indicated by the DSSP analysis (Figs 4a and S4). Interestingly, in one of the simulations the C-terminal region, initially forming the fifth β-strand, adopts a flexible random coil conformation at around 180 ns of the simulation, coincident with dissociation of the region from the central β-sheet. To explore the dynamics of this region further this trajectory was extended to 1 μs in which time repeated events of dissociation and re-association of this C-terminal region were observed (Fig. S5). A subset of the conformations sampled in which the region was detached from the main β-sheet are shown in Fig. 4c. In some frames the C-terminal strand disengages but remains nearby; in others, such as the 185 ns frame, the strand moves far from central sheet (Fig. 4d). This key observation was replicated in a 1 μs trajectory of the same system carried out with the AMBER99SB force field and the TIP3P water model. Although still a relatively rare event, the C-terminal strand repeatedly detached and reattached from the core 3-strand β-sheet (Figs S5 and S6). This trajectory also reproduced the RMSF profiles of the initial MD runs (Fig. 3, dashed line).

The apparent discrepancy between lifetime of secondary structure elements in the MD simulations (strands retained throughout the majority of the simulations, Figs S4 and S5) and the subtle NMR chemical shift deviation from random coil towards β-strand (between 10% and 20% typically, Fig. 4c,d and Table S2) are reflective of the differing sampling rates of the two techniques. The chemical shift measured is an average of the conformational space sampled over micro to milliseconds thus the secondary structure elements visible in the structural models may be averaged over the NMR HSQC timescale.

### Probing the instability of the C-terminal strand

The Aggrescan3D server^34^ was used to investigate how detachment of this C-terminal strand could affect the aggregation potential of the structure (Fig. 4e). Overall, the 185 ns structure following C-terminal detachment showed an increase in amyloid propensity compared to the starting structure with total score values of −2.00 and −29.87 respectively. This difference is larger than that between wild-type β2-microglobulin with low amyloid propensity at neutral pH (−73.54) and its well-characterised amyloidogenic ΔN6 variant which aggregates at neutral pH (−52.32)^34^. Furthermore, the total score of medin following C-terminal detachment (−2.00) is greater than that of the highly amyloidogenic β2-microglobulin variant ΔN6 (−52.32), suggesting an extremely high propensity for medin to self-assemble.

Aggrescan3D showed that the first and third β-strands, along with the C-terminal tail region exhibited increased amyloidogenicity following C-terminal detachment (Fig. 4e). Flexibility observed in the C-terminal region exposes two key regions of the medin sequence that are predicted to be highly amyloidogenic and were previously shown to form fibrils instantly as isolated peptides (residues 32–41 and 42–50)^14^,^35^. Larsson *et al*., also investigated fibrillation of the longer peptide 31–50 which showed slower fibril formation than the smaller peptide fragments, possibly due to rearrangement and dissociation of latent strands that may be present. In addition prediction servers also suggest the N-terminal strand in our model to be amyloidogenic^36^: at the 185 ns time-point, for example, this strand is fully exposed (Fig. 4e).

## Discussion

Medin is a particularly demanding protein to study under physiological conditions since it aggregates in the typical time window and concentrations required for the majority of structural techniques. To address this challenge and obtain a structure of monomeric medin we employed two complementary techniques. Fragment assembly *ab initio* protein modelling was first used to predict a tertiary structure for monomeric medin. This was followed by ^13^C-direct detection NMR to rapidly obtain CSI and RCI information that together revealed secondary structure information in good agreement with the modelling data. We then investigated the stability of the generated structure and proposed a mechanism for nucleation of medin association to form dimers as a nucleation point for fibrillation.

In all models generated from bioinformatic methods and NMR data the key structural elements of a core consisting of three central strands incorporating residues N7-A13, W21-D25 and K30-I36 are well maintained. Other less favored fold predictions still maintain the key strands with differences at the termini suggesting a lack of stable structure at the termini. We utilized MD simulations to probe this hypothesis and to test the stability of the proposed structural model for medin. MD simulations showed that the C-terminus was prone to detachment from the central core which we hypothesize to be required for initiation of amyloidogenesis.

Previous studies investigating the structure of medin using circular dichroism (CD) spectroscopy suggested that medin was a mixture of random coil and β-sheet^37^, with approx. 30% β-sheet^18^,^38^. NMR data presented here also suggests a propensity for β-sheet (Fig. 2c,d). Results from intrinsic disorder prediction servers suggest that medin is predominantly ordered, with only the termini predicted to be disordered Fig. S7. This is consistent with RMSF and RCI data showing increased flexibility in the N-terminus (Fig. 3), and MD simulations suggesting flexibility in the C-terminus (Fig. 4). This is comparable to most human proteins that are reported to have some disordered residues within their terminal regions^39^, with 97% having predicted disorder in the N- or C-terminal residues^40^. We propose that medin has an intrinsic propensity to adopt β-sheet structure, and transiently populates aggregation-prone conformations with a β-sheet core in which the highly amyloidogenic C-terminal residues initiate nucleation and amyloid growth. The transient nature of the strand-detached amyloidogenic conformation is demonstrated throughout the 1 μs MD simulation (Fig. S5) where although β-sheet is the predominant state of the C-terminal residues from 47–49, there are episodes where this strand is completely or partially detached and subsequently reanneals.

**Figure 5:**
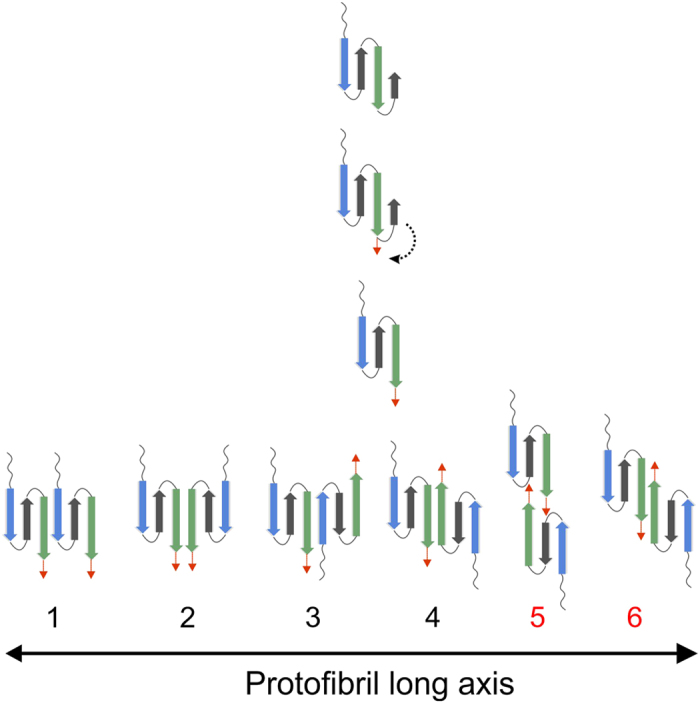
Schematic representation of possible medin dimers. Cartoon illustration of proposed C-terminal strand detachment and subsequent dimer formation through interaction of the amyloidogenic first and third strands (blue and green respectively) as defined by Aggrescan3D (see Fig. 4e). Options 1–4 could involve assembly through pre-formed strands; blue strand (N7-A13) and green strand (K30-I36) with optional involvement of the C-terminal residues 36–50 (red arrow), whereas options 5 and 6 requires stabilisation by the ‘zipping back’ of the C-terminus to form a longer strand encompassing residues 30–50 (green strand and red arrow).

Here we propose that the detachment of the C-terminal strand and subsequent movement away from the central strands may act as the initiating event in self-assembly. Movement of the C- terminal strand opens up possibilities for dimerisation as shown in Fig. 5. We present six arrangements for dimerisation of medin involving association of the key amyloidogenic regions identified above, which maintain the β-sheet arrangement of strand two (W21-D25) and strand three (K30-I36). Four of the orientations assemble *via* strand three (green) pairing with either strand one (blue) or three (green) of a neighbouring molecule in either a parallel or antiparallel orientation (Fig. 5, models 1–4). Multiple studies reveal models for self-association of transthyretin^41^, insulin^42^, mutant β2-microglobulin^43^ and a WW domain from formin binding protein 28^44^ all favour an antiparallel orientation of the same strand (as depicted in model 4, Fig. 5). In the latter work a detachment of one strand from the monomeric structure is required for fibril initiation as posited here for medin.

The detached C-terminal strand provides further options for dimer formation through interaction of this region with the same portion of another monomer (Fig. 5, model 5), or extension of strand three to the terminus (Fig. 5, model 6). Model 5 shares some similarities with a model recently proposed for β2-microglobulin^45^. Model 6 is reminiscent of domain swapped dimers observed for immunoglobulin light chains^46^, prion proteins^47^, β2-microglobulin^48^,^49^ and other proteins^50^. In Fig. 5 dimerisation scenarios are shown with strand three initiating dimerisation, where subsequent monomer/dimer addition would involve strand one self-associating in order to extend the sheet. Although there is no experimental confirmation for which strand initiates dimerisation, several structural observations support our hypothesis.

We propose that the C-terminal region is important for initiating aggregation by first moving away from the sheet to expose key amyloidogenic regions and then engaging in amyloid formation, potentially ‘zipping’ back from the C terminus to extend the β-strand along the entire sequence to include residues 30–50 as shown in the dimer models, Fig. 5. Aromatic interactions are often implicated in amyloid assembly through π-π stacking^51^. The proposed structural model for medin positions the aromatic residues (W11, W22) within the core β-sheet region (Fig. S8) in a manner that would allow for stacking within and between sheets. In addition phenylalanine residues previously implicated in assembly of medin^52^,^53^ are readily accessible within flexible regions (F8 in the flexible N-terminus, F42 and F49 in the extended C-terminus (red region in Fig. 5)), and could assist in driving the extension of the C-terminal β-strand as indicated in Figs 4 and 5. In addition, we recently showed that medin can be nitrated at positions Y16 and W11/W22 with subsequent alterations in aggregation properties^38^. The model proposed here is consistent with all of these sites being accessible for modification altering the ability of the protein to assemble through intermolecular facial β-sheet interactions (Fig. S8).

Furthermore, the models presented here are consistent with formation of a β-hairpin and an extended C-terminal β-sheet as described in our previous fibrillar model for medin using solid state NMR^18^. The fibrillar model was proposed based on solid state NMR data suggesting medin fibrils are composed of a mixture of β-sheet and random coil, the possibility of stabilisation of the hairpin with a salt-bridge between D25 and K30, and an interaction between I35 or I36 and W11 or W21. This study probing soluble medin and early stages in aggregation initiation has autonomously generated models that are consistent with the experimental findings of the previous work on stable mature amyloid-like fibres (see Fig. S8), suggesting that structural rearrangement of the protofibrils could occur to produce mature fibrils with extended β-sheet structure.

We present here the first model for the amyloidogenic protein medin in its transient monomeric form determined using a complementary approach of structural bioinformatics and NMR. The concept of a protective loop shielding aggregation prone regions has also been proposed for other self-assembling proteins^54^,^55^ and as a method of ‘negative design’ to prevent aggregation^56^. This mechanism of the loop moving out of the way to allow dimerisation resembles models previously proposed for several other amyloidogenic proteins suggesting that despite variations in sequence and initial structure, these proteins may share a common pathway for amyloid nucleation and subsequent protofibril and fibril formation.

## Methods

### Structure prediction

A consensus secondary structure prediction for medin, to which 18 different methods contributed, was obtained from the Genesilico Metaserver^57^ (Fig. S1). The structure of soluble monomeric medin was predicted by fragment assembly *ab initio* methods using both the QUARK server^15^ and locally installed ROSETTA^16^,^58^. The QUARK server returns ten models, each a representative of a cluster of similar structures found within the modelled set of predictions. Likewise, 5000 predictions were generated and clustered with ROSETTA, and cluster representatives identified. These cluster representatives are considered candidate fold predictions^59^. The 3- and 9-residue fragment libraries required for ROSETTA were obtained from the ROBETTA server^60^. Libraries of fragments were generated with (NHC) or without the exclusion of homologous sequences i.e. to exclude (NHC) or not fragments of the full length lactadherin C2 domain sequence and homologues. ROSETTA modelling was done with each set of libraries and the results compared. A secondary structure prediction in PSIPRED file format with reliability scores is also required for Rosetta. The prediction from the PSIPRED server^61^,^62^, matching closely the consensus prediction which in turn which agreed well with experimental data, was used. THESEUS^63^ was used for superposition of structures and PyMOL (http://www.pymol.org) for their visualisation. Secondary structure within the models was detected using DSSP^64^ and STRIDE^65^ which agreed exactly in their specification of β-strands. eFOLD^66^ was used to search the Protein Data Bank (PDB)^67^ for structural neighbours of the models.

### Molecular Dynamics

The stability and dynamic properties of the top QUARK model were initially explored using the GROMACS package, release 5.0.6^68^ in conjunction with the OPLS-AA/L all-atom force field^69^. The model was placed in a cubic box of water so that it was at least 1 nm from any edge. With amino acid protonation states assigned for pH 7, the system was neutral so no counterions were added. The SPC/E water model was used^70^. The system was then energy minimised using the steepest descents algorithm. Four independent 200 ns trajectories with different starting velocities were then calculated, with one extended to 1 μs. A further 1 μs trajectory was generated using the AMBER99SB force field^71^ and the TIP3P water model^72^. For long-range electrostatic terms, the Particle-Mesh Ewald (PME) algorithm^73^ was used with non-bonded and van der Waals cut-offs of 1 nm. The LINCS algorithm^74^ was used to constrain all bond lengths. The temperature of the simulations was set to 310.15 K to represent the physiological temperature at which medin aggregation occurs. A pressure of 1bar was used with an integration time step of 2fs and periodic boundary conditions applied. Programs of the GROMACS suite were also used to calculate per-residue, all atom RMSF during the trajectories and to characterise the variation in secondary structure visible through the trajectory by DSSP^64^ assignments.

### NMR methods

^13^C, ^15^N uniform isotope labelled medin was expressed as previously described^38^. Medin concentration was adjusted to 80 μM in 20 mM sodium phosphate (NaPhos), 20 mM sodium chloride (NaCl) pH 6.5, containing 10% ^2^H_2_0 for all NMR experiments. All solution-state NMR experiments were performed at 288 K to reduce the rate of medin aggregation. Two dimensional ^13^C-direct detected (CON and CACO) spectra were acquired on a Bruker AVANCE III 600 MHz spectrometer equipped with an observe 5 mm triple resonance (TXO) cryoprobe optimised for ^13^C detection (Wellcome Trust NMR facility at the University of Birmingham). All other experiments were collected at the University of Liverpool Centre for Structural Biology on a Bruker AVANCE III 800 MHz spectrometer equipped with an inverse 5 mm triple resonance (TCI) cryoprobe. A schematic of the experimental workflow used for backbone assignment is detailed in Fig. 2a. In brief, 2D CON and CACO ^13^C-direct detected experiments^75^ were recorded alongside ^1^H detected 2D experiments to establish ^1^HN, ^13^Cα, ^13^CO, ^1^HCα and ^15^NH assignments. Pulse sequences and parameter sets for these experiments are detailed in Table S1. ^1^H-^15^N Band-selective optimised flip-angle short-transient heteronuclear multiple quantum coherence (SOFAST HMQC) experiments were interleaved throughout to monitor the effect of aggregation as described previously^26^. Fast pulsing methods were not employed for ^13^C-direct detection due to the inherent low sensitivity of the technique. Spectra were processed using Topspin 3.1 (Bruker) and assignment and secondary structure analysis carried out using CCPN software Analysis^29^. The assigned chemical shifts were submitted to CSI 2.0^33^ server to interrogate backbone dynamics. The shift between medin backbone assignment and predicted random coil chemical shift were calculated for C’, Cα and Hα using three reference libraries (Tamiola *et al*.^28^, Wang and Jardetsky^76^, and Schwarzinger *et al*.^77^) within ncIDP and secondary structure propensities calculated using this data and independently from SSP (C’ and Cα shifts)^27^. Propensities were calculated to be 10, 20 or 50% towards either α-helix or β-strand from random coil chemical shift values as reported in Wishart and Sykes^31^ when two out of the three backbone shifts were in agreement. Strands and helices were inferred when three or more consecutive residues have greater than 10% structure propensity. The chemical shifts have been deposited in the BioMagRes-Bank accession No 27021.

## Additional Information

**How to cite this article**: Davies, H. A. *et al*. Probing Medin Monomer Structure and its Amyloid Nucleation Using ^13^C-Direct Detection NMR in Combination with Structural Bioinformatics. *Sci. Rep.* **7**, 45224; doi: 10.1038/srep45224 (2017).

**Publisher's note:** Springer Nature remains neutral with regard to jurisdictional claims in published maps and institutional affiliations.

## Supplementary Material

Supplementary Information
