# Supplementary material for: Probing Medin Monomer Structure and its Amyloid Nucleation Using 13C-Direct Detection NMR in Combination with Structural Bioinformatics
Source: Sci Rep. 2017 Mar 22;7:45224. doi: 10.1038/srep45224 (PMC5361114; doi:10.1038/srep45224)
Supplement: Supplementary Information [file srep45224-s1.pdf]

Supplementary Information

Probing Medin Monomer Structure and its Amyloid Nucleation Using ^{13}C-Direct Detection NMR in Combination with Structural Bioinformatics

Hannah A. Davies, Daniel J. Rigden, Marie M. Phelan and Jillian Madine

Table S1. Detailed parameter sets for all NMR experiments employed in this work. * set by Bruker using constant (cnst) 22.

	EXPERIMENT				
	**SOFAST HMQC**	**^{13}C HSQC**	**H(N)HA**	**CON**	**CACO**
**Pulse programme**	sfhmqcf3gpph	hsqcctetgpsisp	hanhgpwg3d	c_con_iasq	c_caco.ia
**^{1}H spectrometer field (MHz)**	800	800	800	600	600
**Number of scans**	32	16	16	160	64
**Spectral width direct dimension (ppm)**	15.98	13.95	13.95	10.00	40.06
**Spectral width indirect dimension (ppm)**	20.30	20.00	2.60	20.31	19.00
**FID resolution indirect dimension (Hz)**	19.27	36.60	32.51	13.70	14.32
**Direct offset (ppm)**	4.64	4.68	4.71	170.50	54.00*
**Indirect offset (ppm)**	117.65	57.5	4.71	117.65	170.50
**Experiment duration (hours)**	0.25	1.25	0.75	11.5	8

Table S2. Secondary structure propensities obtained from chemical shift data. Change in chemical shift (ΔC’, ΔCα and ΔHα) between medin backbone assignment and predicted random coil chemical shifts calculated using neighbour corrected Intrinsically Disordered Protein Library (ncIDP)[1] and CCPN[2]. ncIDP results are generated using 3 different chemical libraries as shown. Values consistent with β-strand and α-helix are shown in red and blue respectively, shaded according to increasing propensity values. The fourth column for each library reports 10, 20 or 50% deviation towards either α-helix or β-strand from random coil chemical shift values as reported in Wishart and Sykes[3] when two out of the three backbone shifts were in agreement. Residues where three or more consecutive residues have greater than 10% structure propensity have coloured boxes. A graphical representation of the data in column 4 is shown in Figure 2d.

	aa	Tamiola, Acar and Mulder[1]				Wang and Jardetzky[4]				Schwarzinger et al.[5]				CCPN[2]			
		ΔC’	ΔCα	ΔHα		ΔC’	ΔCα	ΔHα		ΔC’	ΔCα	ΔHα		ΔC’	ΔCα	ΔHα	
1	R													-0.15	-1.32	0.14	20β
2	L	-0.52	--	0.38	20β	-0.1	--	0.29		-1.01	--	0.28	50β	-0.93	--	0.3	50β
3	D	-1.07	3.92	0.03		-0.72	4.18	-0.08	20α	0.01	5.27	-0.24	50α	0.09	5.28	-0.23	50α
4	K	--	--	--		--	--	--		--	--	--		--	--	--	
5	Q	-0.17	-0.03	0.11	10β	0.09	0.07	0.07		-0.4	-0.17	-0.04	10β	-0.27	-0.12	-0.02	
6	G	-0.2	0.04	--		-0.42	-0.32	--	20β	-0.63	-0.08	--		-0.33	-0.02	--	
7	N	1.1	0.99	0.04	20α	1.5	1.27	-0.06	20α	0.76	0.9	-0.08	20α	0.88	0.92	-0.07	20α
8	F	-0.63	-0.36	0.09	20β	-0.2	-0.08	0.11	10β	-1	-0.5	0.01	20β	-0.89	-0.47	0.03	20β
9	N	0.5	0.07	0.15		0.09	-0.14	0.08	10β	-0.24	-0.24	0.02	10β	0.05	-0.26	0.11	10β
10	A	-0.62	0.01	0.06	10β	0.29	0.12	-0.05	10α	-0.5	-0.16	-0.03	10β	-0.2	-0.12	0.03	
11	W	2.11	0.31	0.03	10α	0.14	0.18	-0.07		-0.39	0.31	-0.09	10α	-0.25	0.32	-0.06	10α
12	V	-0.26	-0.22	0.12	10β	-0.02	-0.12	-0.13		-0.7	-0.51	-0.06	20β	-0.67	-0.53	-0.06	20β
13	A	-0.11	0.33	-0.16	10α	0.36	0.58	-0.26	20α	-0.21	0.22	-0.27		0.04	0.28	-0.1	10α
14	G	-0.28	-0.02	--		-0.31	-0.14	--	10β	-0.49	-0.15	--	10β	-0.18	-0.08	--	
15	S	-0.47	0.05	0.15	10β	0.1	0.21	0.07		-0.63	-0.23	0.09	20β	-0.61	-0.21	0.09	20β
16	Y	-0.08	0.11	0.01		0.42	0.16	-0.02		-0.47	-0.4	-0.06	20β	-0.38	-0.32	-0.05	20β
17	G	0.59	0.39	--	10α	-0.01	-0.02	--		-0.26	-0.12	--		-0.12	-0.08	--	
18	N	0.02	0.22	0.07		0.35	0.34	0.02	10α	-0.53	0.06	-0.09		-0.35	0.06	-0.04	
19	D	-0.07	0.23	0.02		0.4	0.21	-0.03		0.99	1.59	-0.27	50α	1.25	1.62	-0.19	50α
20	Q	-0.18	0.55	0.02		0.21	0.45	-0.06	10α	-0.12	0.2	-0.09		0.04	0.18	-0.04	
21	W	2	-0.21	0.11	10β	-0.22	-0.54	0.07	10β	-0.64	-0.31	-0.01	20β	-0.55	-0.28	0.01	20β
22	L	-0.44	-0.11	0.18	20β	0.24	-0.07	-0.03		-0.84	-0.4	0.02	20β	-0.61	-0.31	0.04	20β
23	Q	-0.08	0.01	0.03		-0.15	-0.15	-0.08	10β	-0.45	-0.3	-0.06	20β	-0.2	-0.21	0.12	10β
24	V	-0.44	-0.14	0.09	10β	-0.29	-0.17	-0.02	10β	-0.91	-0.4	-0.06	20β	-0.77	-0.42	-0.01	20β
25	D	0.09	-0.23	0.13	10β	0.21	0.05	0		1.05	1.11	-0.13	20α	1.08	1.11	-0.13	20α
26	L	0.08	0.04	0.06		0.94	0.5	0	10α	0.11	-0.17	-0.06		0.22	-0.15	-0.04	10α
27	G	0.29	0.42	--	10α	0.08	0.28	--		-0.05	0.24	--		0.07	0.26	--	
28	S	-0.14	0.16	0.08	10β	0.19	0.32	-0.01	10α	-0.39	-0.26	-0.03	10β	-0.3	-0.24	0	10β
29	S	-0.1	0.19	0.08		0.31	0.16	0.04		-0.26	-0.17	-0.02	10β	-0.17	-0.13	0	
30	K	-0.22	0.13	0.06	10β	0.18	-0.02	0.05		-0.4	-0.12	-0.04		-0.14	-0.06	-0.01	
31	E	0.01	-0.23	0.12	10β	0.21	-0.24	0		0.31	0.53	-0.07	10α	0.45	0.55	-0.05	20α
32	V	-0.14	0.05	0.17	10β	0.47	0.42	0	10α	-0.34	-0.16	0.01	10β	-0.31	-0.19	0.01	10β
33	T	-0.07	0.07	0.11		-0.4	0.81	-0.09	20α	-0.57	0.09	-0.05		-0.34	0.14	-0.02	
34	G	-0.35	-0.06	--		-0.26	-0.17	--	10β	-0.6	-0.13	--		-0.37	-0.06	--	
35	I	-0.09	0.05	0.08		0.91	0.61	-0.09	20α	-0.27	-0.23	-0.03	10β	-0.14	-0.24	-0.01	10β
36	I	-0.21	-0.04	0.1	10β	0.59	0.34	-0.05	10α	-0.41	-0.55	0.01	20β	-0.36	-0.55	0.02	20β
37	T	-0.27	0.05	0.13	10β	-0.73	0.27	-0.08	10α	-0.68	-0.14	-0.04	10β	-0.66	-0.17	-0.03	10β
38	Q	-0.16	0.2	0.06	10β	0.07	0.12	0.05		-0.45	-0.14	-0.07	10β	-0.32	-0.16	-0.04	10β
39	G	-0.08	0.04	--		-0.55	-0.27	--	10β	-0.37	-0.06	--		-0.26	-0.05	--	
40	A	-0.1	0.2	0.08		0.34	0.15	-0.04	10α	-0.32	-0.19	-0.04	10β	-0.2	-0.16	-0.03	10β
41	R	-0.45	-0.2	0.08	20β	-0.33	-0.43	0.03	20β	-0.78	-0.55	-0.1	20β	-0.51	-0.46	-0.04	20β
42	N	0.09	-0.07	0.1		0.23	-0.02	0.03		-0.3	-0.12	0		-0.28	-0.16	0	10β
43	F	-0.24	0.02	0		0.34	0.49	0.04		-0.42	-0.08	-0.06		-0.31	-0.09	-0.05	
44	G	0.36	0.37	--	10α	-0.14	-0.11	--		-0.42	-0.09	--		-0.19	-0.02	--	
45	S	-0.19	0.1	-0.03		0.14	0.25	-0.16		-0.45	-0.25	-0.12	10β	-0.3	-0.21	-0.07	10β
46	V	-0.4	-0.08	0.15	20β	0.03	-0.16	0.04	10β	-0.68	-0.35	-0.01	20β	-0.41	-0.29	0.05	20β
47	Q	-0.36	-0.1	0.1	20β	-0.47	-0.12	-0.01		-0.73	-0.38	0.03	20β	-0.47	-0.33	0.06	20β
48	F	-1.08	-4.65	-0.02	50β	-0.98	-4.45	-0.08	50β	-1.38	-4.74	-0.07	50β	-1.24	-4.72	-0.04	50β
49	V	-1.14	0.09	0.09	20β	-1.43	0	-0.07		-1.84	-0.4	-0.03	20β	-1.81	-0.42	-0.03	20β

```
             1........10........20........30........40........
             RLDKQGNFNAWVAGSYGNDQWLQVDLGSSKEVTGIITQGARNFGSVQFVA
HMMSTR       EE-----EEEEEE----HHHHEE--HHH-HEEEEEEEE-------EEEEE
sspro4       E---------EE---------EEEEEEEEEEEEEEEEE-EEE--EEEEE-
sspal        HH-----EEEEEE-------EEEEE-----EEEEEEE--------EEEEE
jnet         -------E-----------EEEEEE-----EEEEEEEHHHHHHH------
proteus      ---------EEEE------EEEEEEE-EEEEEEEEEEEEE-----EEEEE
*SPARROW     ----------E----------EEEE----EEEEEEEE-----------E-
sspred       -------------------EEEEEEE--EEEEEEEEEE---EEE--EE--
SPARROW      -----------E---------EEEE----EEEEEEEEE---------E--
nnssp        ---------EEEE-------EEEEE------EEEEEE---------EEEE
netsurfp     --------------------EEEEE----EEEEEEEEE------------
ssp          --------------------EEEEEEE---EEEEEEE-------------
pssfinder    -------EEEEEE--------EEEE----EEEEEEEEE------------
raptorxss    --------------------EEEE-----EEEEEEEE-------------
psspred      ----------E----------EEEE----EEEEEEEE----------E--
spineX       ---------EEE--------EEEEE---EEEE-EEEEE----------E-
spine        --------------------EEEEEE---EEEEEEEE----------E--
psipred      ---------EEE--------HHHHHH--EEEEEEEEE-------------
soprano      --------------------EEEEE----EEEEEEEEE------------
consensus    ----------EE--------EEEEE----EEEEEEEEE---------E--
```

Figure S1. Secondary structure prediction results for human medin obtained using the Genesilico Metaserver [6] and the resulting consensus prediction (bottom row). Green E characters represent predicted β-structure, red H indicates predicted α-helix.

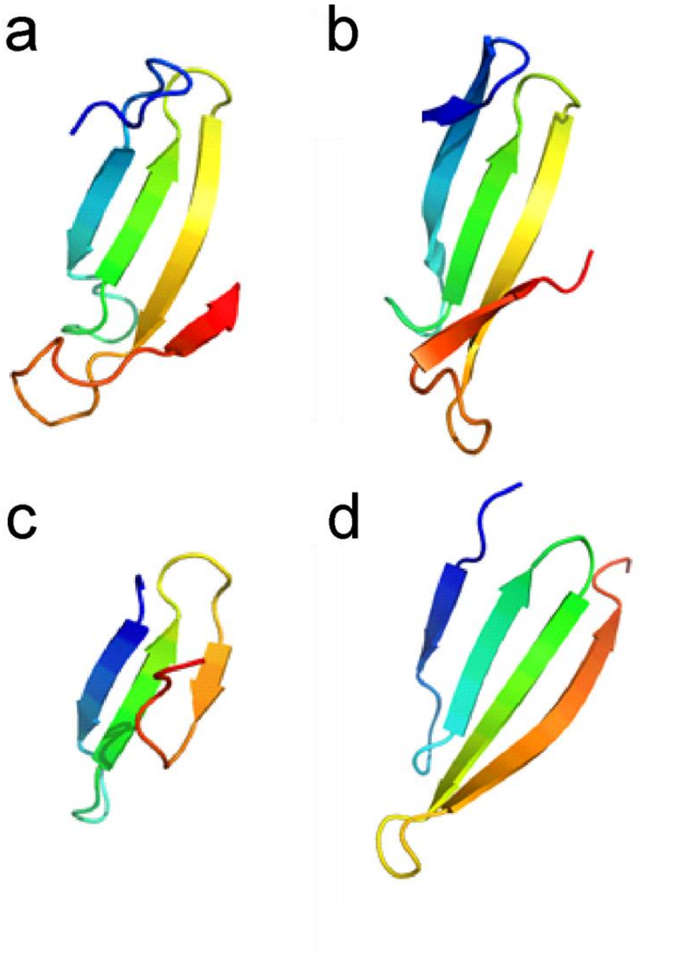

Figure S2. Structure comparison of *ab initio* models of human medin and their respective nearest structural neighbours identified using eFOLD [7]. (a) The medin model obtained from QUARK, (b) the top ROSETTA model (obtained using homologous fragments), (c) WW domain from human PRPF40A (PDB code 2dyf; unpublished) and d) *Bacillus subtilis* YmzC protein (PDB code 3kvp; unpublished). Each structure is coloured from blue (N-terminus) to red (C-terminus).

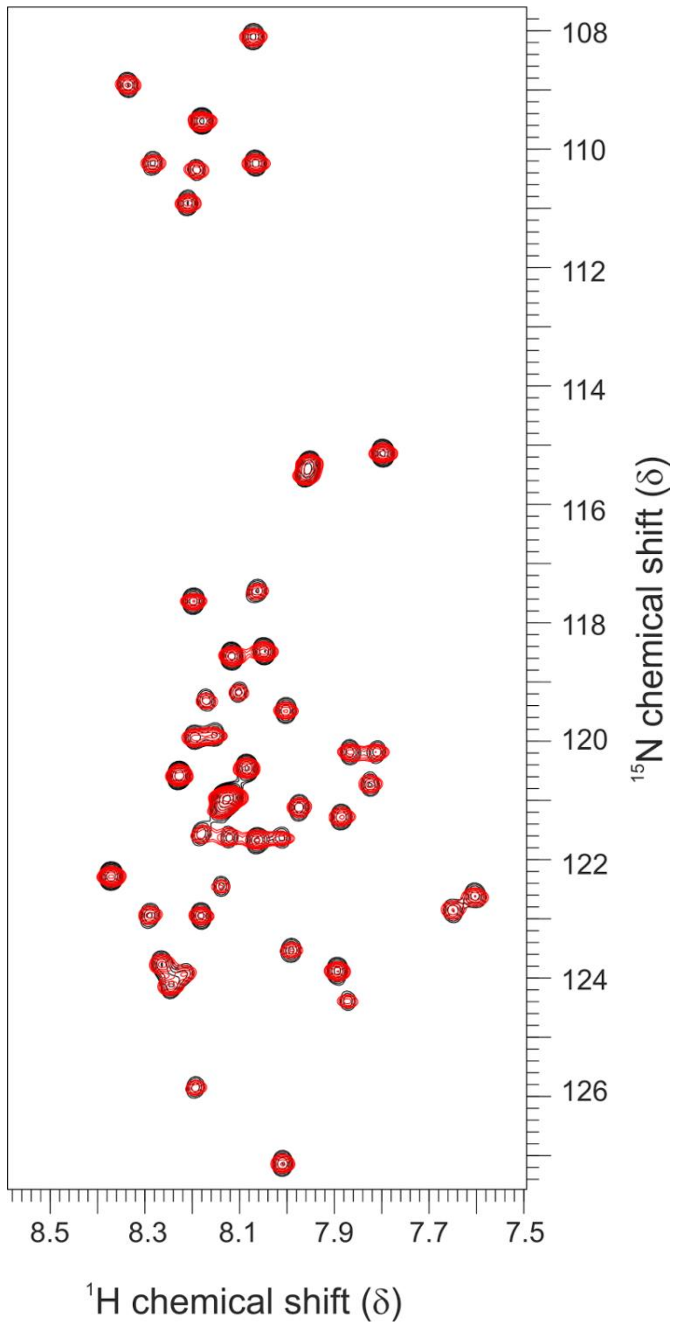

Figure S3. Overlay of ^{1}H-^{15}N SOFAST HMQC spectra of medin at the start (black) and end (red) of assignment experiments showing negligible change over the time course of experiments.

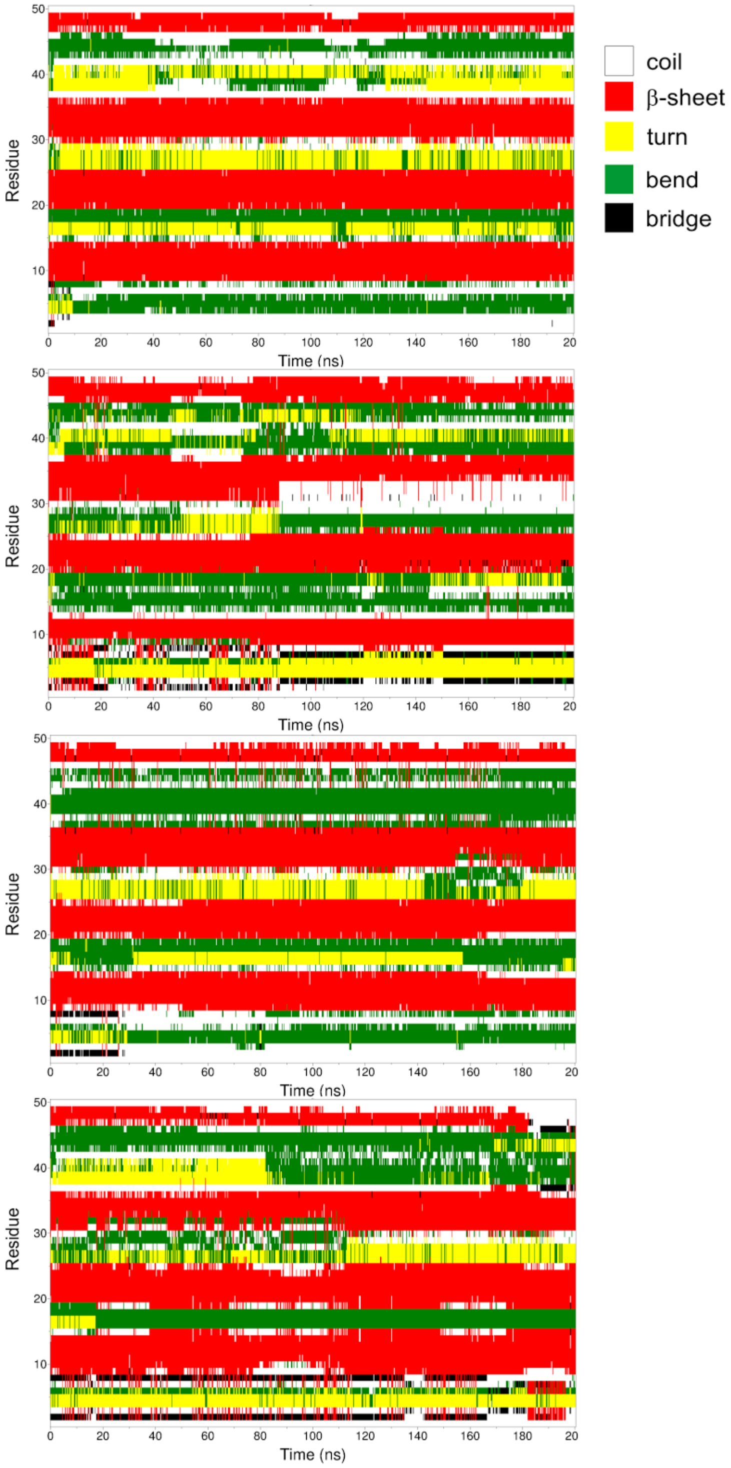

Figure S4. Comparison of the secondary structure matrix of four replicate molecular dynamics trajectories over 200ns. Snapshots each 250ps are color-coded according to secondary structure as assigned by DSSP.

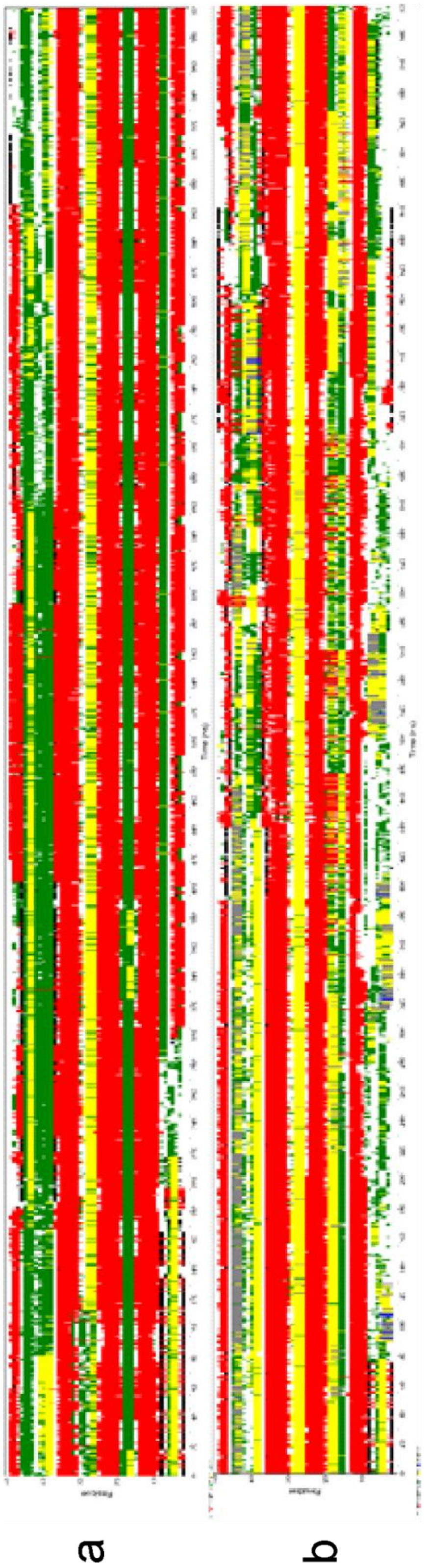

Figure S5. Secondary structure matrix of extended 1μs molecular dynamics trajectories. (a) with the OPLS-AA/L all-atom force field with SPC/E water model and (b) AMBER99SB force field and the TIP3P water model. Snapshots each 250ps are color-coded according to secondary structure as assigned by DSSP. This simulation demonstrates the transient nature of the C terminal strand where β-sheet (red) is the predominant state of residues from 47-49, with episodes of detachment exhibited by an absence of assigned secondary structure (white), and isolated β-bridges (black).

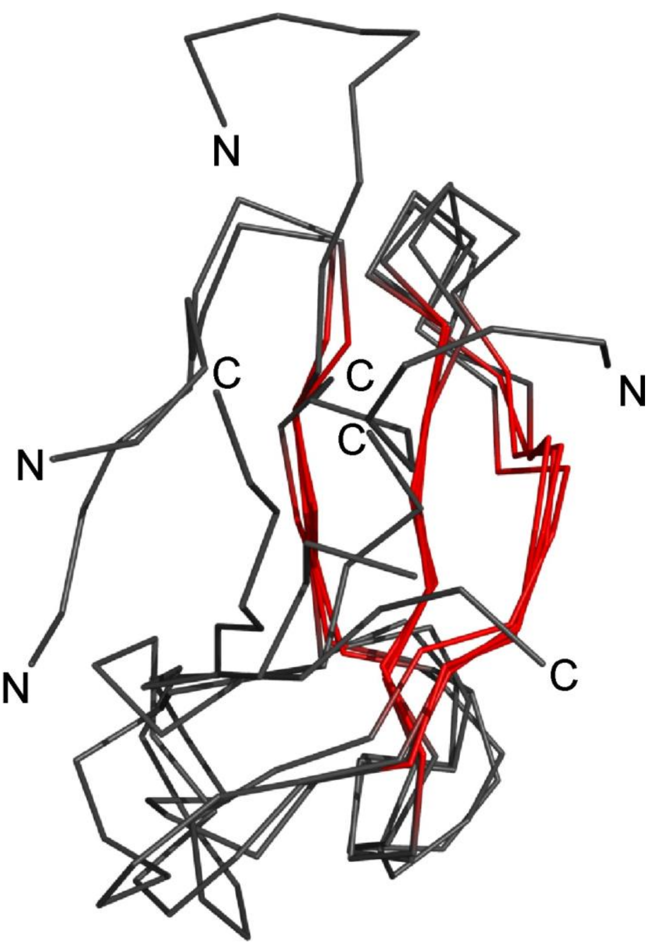

Figure S6. Ensemble of structures from the second MD simulation shown in Figure S6b in which the C-terminal strand is detached from the central sheet.

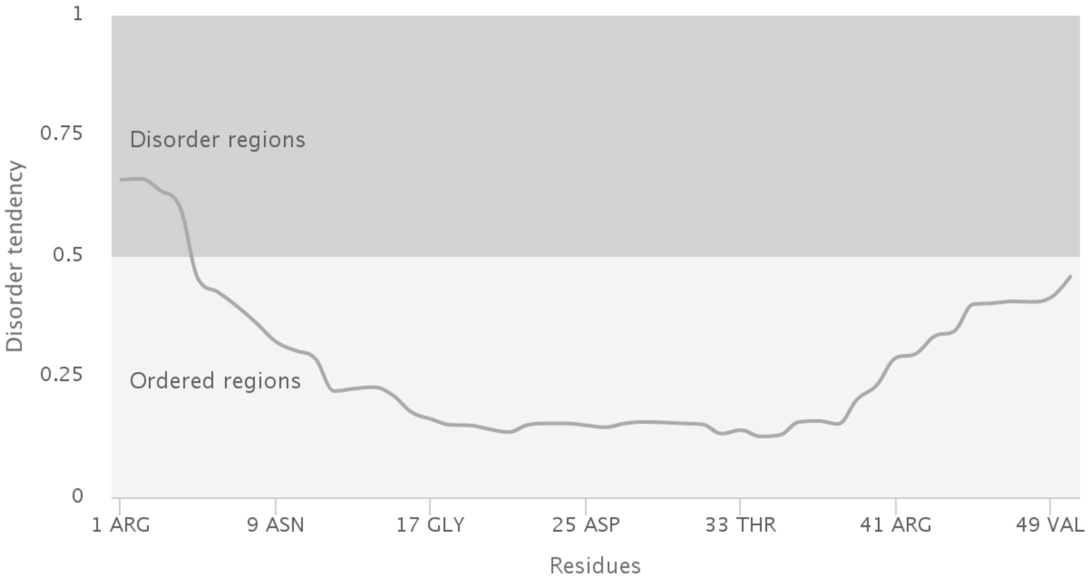

Figure S7. Disorder prediction using MetaDisorderMD2 representing a consensus result based on 13 disorder predictors [8].

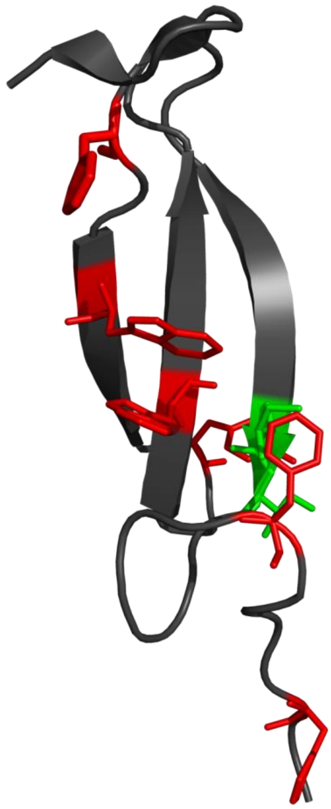

Figure S8. Cartoon representation of the 185ns snapshot highlighting the position of aromatic residues (red sticks) and isoleucine 35 and 36 described in our previous work [9] (green sticks) within model.

Supplementary References

1 Tamiola, K., Acar, B. & Mulder, F. A. A. Sequence-specific random coil chemical shifts of intrinsically disordered proteins. *J. Am. Che. Soc.* **132**, 18000-18003, (2010).

2 Vranken, W. F. *et al.* The CCPN data model for NMR spectroscopy: development of a software pipeline. *Proteins* **59**, 687-696, (2005).

3 Wishart, D. S. & Sykes, B. D. Chemical shifts as a tool for structure determination. *Methods Enzym.* **239**, 363-392, (1994).

4 Wang, Y. & Jardetzky, O. Probability-based protein secondary structure identification using combined NMR chemical-shift data. *Protein Sci.* **11**, 852-861, (2002).

5 Schwarzinger, S. *et al.* Sequence-dependent correction of random coil NMR chemical shifts. *J. Am. Chem. Soc.* **123**, 2970-2978, (2001).

6 Kurowski, M. A. & Bujnicki, J. M. GeneSilico protein structure prediction meta-server. *Nucleic Acids Res.* **31**, 3305-3307, (2003).

7 Krissinel, E. & Henrick, K. Secondary-structure matching (SSM), a new tool for fast protein structure alignment in three dimensions. *Acta Crystallogr. D Biol. Crystallogr.* **60**, 2256-2268, (2004).

8 Kozlowski, L. P. & Bujnicki, J. M. MetaDisorder: a meta-server for the prediction of intrinsic disorder in proteins. *BMC Bioinformatics* **13**, 1-11, (2012).

9 Davies, H. A., Madine, J. & Middleton, D. A. Comparisons with amyloid-beta reveal an aspartate residue that stabilizes fibrils of the aortic amyloid peptide medin. *J. Biol. Chem.* **290**, 7791-7803, (2015).
